# High Throughput Sequencing Technologies as a New Toolbox for Deep Analysis, Characterization and Potentially Authentication of Protection Designation of Origin Cheeses?

**DOI:** 10.1155/2019/5837301

**Published:** 2019-11-20

**Authors:** Elena Kamilari, Marios Tomazou, Athos Antoniades, Dimitrios Tsaltas

**Affiliations:** ^1^Cyprus University of Technology, Department of Agricultural Sciences, Biotechnology and Food Science, Cyprus; ^2^Stremble Ventures Ltd, Cyprus

## Abstract

Protected Designation of Origin (PDO) labeling of cheeses has been established by the European Union (EU) as a quality policy that assures the authenticity of a cheese produced in a specific region by applying traditional production methods. However, currently used scientific methods for differentiating and establishing PDO are limited in terms of time, cost, accuracy and their ability to identify through quantifiable methods PDO fraud. Cheese microbiome is a dynamic community that progressively changes throughout ripening, contributing via its metabolism to unique qualitative and sensorial characteristics that differentiate each cheese. High Throughput Sequencing (HTS) methodologies have enabled the more precise identification of the microbial communities developed in fermented cheeses, characterization of their population dynamics during the cheese ripening process, as well as their contribution to the development of specific organoleptic and physio-chemical characteristics. Therefore, their application may provide an additional tool to identify the key microbial species that contribute to PDO cheeses unique sensorial characteristics and to assist to define their typicityin order to distinguish them from various fraudulent products. Additionally, they may assist the cheese-makers to better evaluate the quality, as well as the safety of their products. In this structured literature review indications are provided on the potential for defining PDO enabling differentiating factors based on distinguishable microbial communities shaped throughout the ripening procedures associated to cheese sensorial characteristics, as revealed through metagenomic and metatranscriptomic studies. Conclusively, HTS applications, even though still underexploited, have the potential to demonstrate how the cheese microbiome can affect the ripening process and sensorial characteristics formation via the catabolism of the available nutrients and interplay with other compounds of the matrix and/or production of microbial origin metabolites and thus their further quality enhancement.

## 1. Introduction

In order to protect traditional products and secure potential economic benefits for local producers, differentiating them from potential commercial fraud, the European Union (EU) has established three types of quality labels: Protected Designation of Origin (PDO), Protected Geographical Indication (PGI) and Traditional Speciality Guaranteed (TSG) (European Commission, 2013) [1]. PDO label refers to agricultural products or foodstuffs, whose production, processing and preparation has been conducted within defined geographical region, following traditionally applied processes. In cheese-making, it has been applied also as a contributing factor of consumers' preference for cheeses in market as well as a matrix to provide food safety [2].

Several methodologies have been used to characterize PDO cheeses, mainly based on their organic compounds and other basic components, including gas chromatography (GC) [3], high-performance liquid chromatography (HPLC) [4, 5], infrared (IR) spectroscopy [6, 7], solid phase microextraction (SPME) and purge and trap (P&T) [8] and nuclear magnetic resonance (NMR) spectroscopy [9, 10]. In addition, as it was indicated, the specific production conditions within the PDO area select for particular microbial populations, which contribute through the fermentation of the available nutrients in the sensorial and safety characteristics of the cheese, introducing the cheese microbiome as a possible fingerprint of cheese authentication [11].

The identification of the microbial communities guiding the fermentation process in different cheeses has been largely attained from traditional culture-dependent, as well as culture-independent molecular methods. The latter methodologies include the analysis of 16S rRNA gene, via denaturing or temperature gradient, gel electrophoresis (DGGE/TGGE) [12–14], fluorescent *in situ* hybridization (FISH) [15], single-stranded conformation polymorphisms (SSCP) [16], terminal-restriction fragment length polymorphism (T-RFLP) [17] or length heterogeneity-PCR (LH-PCR) [18]. More recently, High Throughput Sequencing (HTS) methodologies, in particular amplicon sequencing, shotgun metagenomic sequencing and metatranscriptomics, have being utilized for deeper identification of the highly diverse microbiome present in a cheese's ecosystem as well as for assessing how environmental conditions regulate the gene expression in the community [19, 20]. There are two commonly applied NGS technologies in metagenomic studies, the 454 Life Sciences and the Illumina systems, which allow a quicker and more elaborative microbial genetic pool analysis, at an acceptable cost. Metagenomics, a powerful culture-independent genomic technology, was demonstrated able to identify all microorganisms existing in a cheese sample and their relative abundance, by evaluating their specific DNA sequences [21, 22]. Metatranscriptomics, another culture-independent genomic analysis, was similarly applied to characterize the microbial populations of cheese samples regarding their expressed messenger RNA (mRNA), in order to reveal the functional characteristics of the microbial metabolic processes in cheeses [23].

The producers of numerous PDO labeled cheeses, or of other local cheeses that want to establish denomination of origin, have applied HTS methodologies to characterize their unique microbial profiles as differentiating factor. The latter, aimed at reproducing the desired original sensorial characteristics by identifying and inoculating with selected microorganism cultures during the fermentation process [24–31]. Additionally, the analysis of the microbial biodiversity has important value, not only for understanding the contribution of key microbes in the process of ripening, but also to comprehend how the technological parameters applied influence the quality and safety of the product [32, 33]. The current review focuses on the application of HTS technologies as a new additional tool for establishing of the authenticity of PDO cheeses. These technologies may allow the identification of the microbial communities developed in PDO cheeses, to explore the contribution of manufacturing conditions as well as to uncover additional factors that contribute to microbial communities' formation and also to investigate the influence of the metabolically active microbiota in cheese sensorial characteristics development.

## 2. Understanding the Cheese Microbiome via HTS

### 2.1. Introduction to Cheese Microbiome

Each type of cheese is comprised by a unique pool of microbes. This pool includes the dominant population (the lactic acid bacteria), favored by the configured microenvironment and consequently colonizing the cheese more successfully, the subdominant, which are microbes found in lower abundance due to the restrictive pressure of the dominant species, and finally the low abundant species group that exists in the cheese mostly due to contamination from the environment [34]. The initial dominance of Starter Lactic Acid Bacteria (SLAB) is based on their metabolic capacity to successfully ferment lactose. For long ripened cheeses, however, other Non-Starter Lactic Acid Bacteria (NSLAB), with elevated proteolytic and lipolytic ability and able to metabolize other carbon sources than lactose, are capable to dominate cheese microenvironments affecting cheeses' flavor and texture [34].

*Lactococcus lactis* and *Streptococcus thermophilus* are among the dominant species and are often used as starters in cheese industry contributing to the development of cheese sensorial characteristics [35, 36]. Members of the genus *Lactobacillus,* including *Lactobacillus delbrueckii* are also used as starters in dairy products production. Other species, such as *Lactobacillus casei*, *Lactobacillus paracasei, Lactobacillus helveticus*, *Lactobacillus plantarum/paraplantarum, Lactobacillus brevis* and *Lactobacillus rhamnosus,* although they are rarely applied as starters, are also vital contributors to cheese organoleptic characteristics formation. Other LAB, including some enterococci (mostly *E. faecalis, E. faecium* and/or *E. durans)* and *Leuconostoc* (mostly the species *Leuconostoc mesenteroides* and *Leuconostoc pseudomesenteroides*) are additional starters [37, 38]. Additional LAB bacteria, including members of the genera *Streptococcus*, *Pediococcus* and *Weissella,* as well as members of the genera *Corynebacterium*, *Arthrobacter*, *Brevibacterium*, *Propionibacterium* could also be detected in lower relative abundances [35]. Spoilage and pathogenic bacteria that are frequently detected in cheese, involve the genera *Clostridium*, including *Clostridium tyrobutyricum, Clostridium perfringens* and *Clostridium butyricum*, *Staphylococcus*, including *Staphylococcus aureus,Salmonella* spp*., Listeria* spp*., E. coli,Shiggella* spp., *Bacillus cereus, Pseudomonas aeruginosa*, *Citrobacter freundii,Klebsiella pneumoniae*, andmembers of the genera *Enterobacter*, *Psychrobacter, Proteus*, *Serratia* and *Halomonas.* The cheese microbiome is also comprised of yeast, such as *Kluyveromyces lactis*, *Candida* spp., *Debaryomyces hansenii, Yarrowia lipolytica* and *Geotrichum candidum,* and moulds including members of the genera *Geotrichum, Penicillium*, *Mucor*, *Aspergillus*, and *Fusarium* [39].

### 2.2. HTS Technologies Employed in Microbial Research

HTS technology is widely applied to study whole microbial communities in numerous microenvironments. The three major approaches used, include:

  (a) Amplicon sequencing, according to which by targeting a particular conserved region, a specific gene, gene fragment, as well as sequence could be amplified and the sequence to be recognized,
  (b) Shotgun metagenome sequencing, whereby the whole DNA of a specimen is sequenced to specify existing genes and.
  (c) Metatranscriptome sequencing (RNA-seq), in which the total amount of transcripts in a specimen is sequenced, enabling the identification of the relative gene expression activity of the members within the microenvironment analyzed [40].

The basic differences among these approaches include the library preparation process, the sequencing practice and the bioinformatic analysis. Detailed analysis of the currently available platforms, including their advantages and disadvantages, has been extensively reviewed [36, 41–43]. The application of high-throughput sequencing has improved the amount and quality of information extracted from microbiological analysis, allowing the discrimination of the basic, highly abundant species that dominate the cheese microbiome from less represented microbes (Table 1), with detection sensitivities and throughputs a great number of times higher, compare to previously used molecular techniques [42].

**Figure 1:**
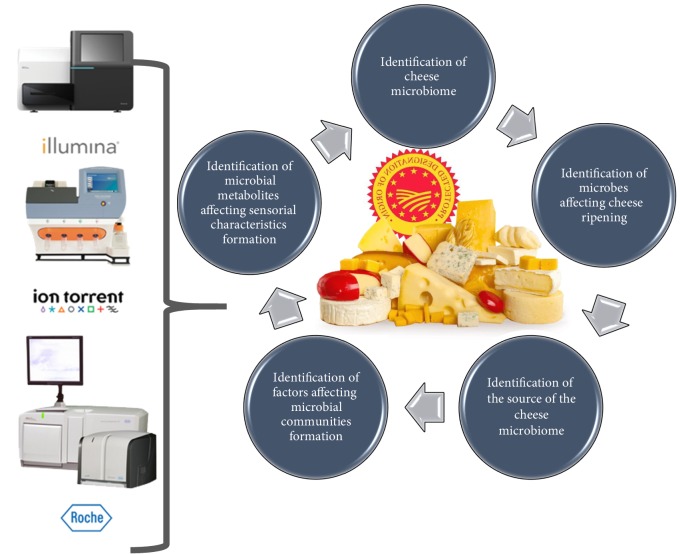
Application of HTS methodologies for the characterization of the cheese microbiome and its dynamics throughout ripening as well as its influence in sensorial characteristics formation.

The use of HTS technologies as a powerful tool for characterizing the cheese microbial composition and its dynamics has been demonstrated through several recent studies (Figure 1). The findings and level of characterization from these studies as discussed in the following sections, strongly support that HTS mediated microbial fingerprinting can become an important toolbox for establishing PDO in cheeses.

#### 2.2.1. HTS Analysis for the Identification of the Cheese Microbiome

HTS methodologies have been applied recently to identify the microbiome of several traditional and PDO cheeses [23, 25–27, 44–46]. Pico cheese, a traditional PDO cheese produced in Azores (Portugal), was analyzed via high throughput sequencing to uncover the core microbiome responsible for providing the unique sensorial characteristics that classify Pico cheese among gourmet products, to improve its marketability and to guarantee its safety for consumers [28]. Although significant differences were detected in the relative abundance of the bacteria among the different cheese makers and maturation times, Operational Taxonomic Unit (OTU) analysis revealed the presence of common genera, including *Acinetobacter, Enterococcus, Lactobacillus, Lactococcus, Leuconostoc, Pantoea, Rothia, Staphylococcus, Streptococcus* and unclassified genera members of the Enterobacteriaceae family, in all the samples analyzed. *Lactococcus* was the dominant genus, highlighting their importance for Pico cheese' sensorial characteristics development [28]. 16S rRNA metagenomic sequencing analysis revealed that the core microbiome of traditional Buryatian cottage cheese was composed mainly by *Lactococcus*, followed by *Streptococcus*, *Pseudomonas*, *Acetobacter,Klebsiella*, *Lactobacillus,Acinetobacter* and *Raoultella* [47]. Serpa is another traditional ripened Portuguese cheese that was granted the Protected Designation of Origin (PDO) label. Investigation of the yeasts' communities in Serpa cheeses via HTS, has revealed 11 main genera (*Cryptococcus, Hanseniaspora, Saccharomyces, Kluyveromyces, Debaryomyces, Pichia, Metschnikowia, Yarrowia, Candida, Galactomyces, Penicillium*), as well as two species, *Debaryomyces hansenii* and *Kluyveromyces marxianus*, among the most commonly detected, high-abundance taxa. Differences in the fungal communities of the PDO registered compare to non-PDO registered industries were reported [31]. Furthermore, traditionally-aged cheeses, shape a dynamic microbial community on their surface (rind) as they age. The rind biofilm, is a complex network of interacting bacterial and fungal species, the identification of which is considered important for cheesemakers, who sometimes deal with undesirable growth of microbes on cheese surface or unwanted microbes that interfere with the ripening process [48]. HTS analysis of a collection of 137 different rind samples in traditionally-aged cheeses from 10 countries, uncovered the existence of a core microbial pool, highly reproducible, composed of 24 dominant genera, 14 bacterial and 10 fungal. Two firstly detected in foods bacterial genera, *Yaniella* and *Nocardiopsis*, were identified, as well as some halotolerant *γ*-Proteobacteria, including the genera *Vibrio, Halomonas* and *Pseudoalteromonas*, which were present in all cheeses tested, independently of their geographic origin [48].

#### 2.2.2. HTS Allows the Investigation of the Temporal Distribution of Microbes Throughout Ripening

Metagenomic analysis may be applied to investigate dynamic changes in the microbial communities that take place during cheese ripening. Analysis of a possible microbiological signature of the French “Tomme d'Orchies” during the ripening procedure classified *Lactococcus* spp. and *Streptococcus* spp. as the dominant genera that comprised the core microbiome of the cheese [49]. At the beginning of ripening, *Lactococcus* spp. were the dominant fermenters (66.09%) and *Streptococcus* spp. the second most abundant (29.47%). At the end of the ripening process the relative abundance of *Lactococcus* spp. was drastically reduced (8.80%) whereas of *Streptococcus* spp. was severely increased (88%). 16S rRNA sequencing of PDO Silter raw milk cheese bacterial communities indicated that the addition of an autochthonous starter culture increased the relative abundance of LAB and reduced the harmful bacteria, without affecting the typical microbiome of Silter cheese [50].

#### 2.2.3. HTS Reveals a Broader Microbial Diversity in Cheese Compared to Traditional Methods

HTS analysis identified the presence of species that their existence was unknown, either because are challenging to culture, or found in limited abundance. For instance, such rare microbes belong to the genera *Pseudoalteromonas, Facklamia, Vibrio* and the species *Geobacillus toebii* and *Methylobacterium populi* [51, 52]. Moreover, metagenomics studies indicated the presence of genera found in animals' gut, but never reported in cheese samples before, such as the genera *Prevotella* and *Faecalibacterium* or detected genera in particular types of cheeses for the first time, including the genera *Arthrobacter* and *Brachybacterium* [51]. The specific bacteria which contribute to the unique sensorial characteristics of the Mexican Cotija cheese were defined by HTS [21]. The analysis recognized more than 500 taxa, from which the most dominant were *L. plantarum*, *L. mesenteroides* and *W. paramesenteroides* (80%). The increased relative abundance of *Weissella* and *Leuconostoc* species, suggested a possible unique characteristic microbial signature of the authentic ripened Cotija cheese. Among the low abundant species, some halophilic bacteria and archaea were also reported. Interestingly, pathogenic bacteria were absent despite the presence of genes expressing bacteriocins.

### 2.3. HTS Analysis Reveals the Source of Fermented Cheeses Microbiome

The complex and dynamic microbial associations developed in fermented cheeses are influenced by a combination of factors shaping the cheese making process. The factors affecting the microbial community formation have been analyzed extensively in another review [53]. By discussing the advantages of HTS technologies and their potential applications on analyzing the cheese microbiome and uncovering its contribution to cheese sensorial characteristics, the authors highlighted the importance of applying HTS technologies in cheese industry. As mentioned, some of these fluctuations in the relative abundance of microbes arise from the origin of milk and the cheese manufacturing environment, including the addition of natural whey or other starting cultures, salt, herbs or other ingredients and whether the milk is raw or pasteurized [24, 34, 51, 54, 55]. In the case of PDO cheeses there are specific guidelines that the producers are obliged to follow during cheesemaking. However, the lack of consistent traceability systems cannot differentiate the PDO cheeses from fraud products. Since the environmental and production conditions are core components of PDO, HTS methodologies can support further the authentication by identifying the microbial imprint of these factors on the cheese of interest. Some of the factors that need to be evaluated are discussed below.

#### 2.3.1. Starter Cultures

Among the major sources from where the formation of microbial populations in fermented cheeses originate are starter cultures. Traditionally, the use of natural whey cultures (NWC) has been applied in curd fermentation, as a source of thermophilic lactic acid bacteria (LAB). A number of PDO cheeses, including Asiago Pressato, Gorgonzola, Asiago d'Allevo, Quartirolo Lombardo, Taleggio, Raschera Montasio, as well as Mozzarella cheese in Italy, use NWC as starting cultures [56]. In various PDO cheeses, including Parmigiano Reggiano, Grana Padano, Provolone Valpadana, Mozzarella di Bufala Campana and Caciocavallo Silano, the addition of NWC was found to be essential for cheese flavor development [57]. NWC are created by heating the raw milk at 60–63°C, for 20–30 min, and then incubating it at temperatures 39–42°C. 16S rRNA sequencing analysis revealed that NWC were mostly dominated by *Streptococcus thermophilus* and *Lactobacillus delbrueckii* [54, 56, 58]. Mozzarella, one of the most familiar non-ripened cheese, is manufactured in southern Italy basically using whole raw water buffalo's milk, with the addition of NWC. Pyrosequencing analysis of the bacterial communities found in raw milk, NWC and curd at the beginning and end of the ripening, as well as final traditional mozzarella cheese from two dairies, showed that the NWC starter was the source of bacteria implicated in the fermentation and not the bacteria existed in raw milk [45]. The importance of the contribution of NWCs for curd fermentation was indicated in an additional study for Mozzarella cheese and two other Italian PDO cheeses, the Grana Padano and Parmigiano Reggiano [26]. Although a species-based distinction was observed among the three cheeses, as revealed via HTS of lacS gene amplicons of *S. thermophilus* species, all of them had the same core microbiome, comprised of *S. thermophilus*, *L. lactis* and *L. helveticus,L. delbrueckii andL. fermentum* originated by the NWC. The microbial community of Poro cheese was also originated mostly by fermented whey and represented similarly mostly by the species *Streptococcus salivarius* subsp. *thermophilus* and *Lactobacillus delbrueckii* [25].

Animal rennet comprises also a source of potential starter LAB, including *S. thermophilus* and some lactobacilli, mainly *Lactobacillus crispatus* and *Lactobacillus reuteri* as revealed via HTS analysis [59]. Animal rennets have been utilized for years in cheese making process as coagulant agents, after their isolation from abomasum of ruminants, mostly lamb veal and kid. The isolation, identification and characterization of the microbial strains that dominate NWC and NMC, responsible for cheese ripening, has allowed their use as starter cultures. Indeed, numerous traditional artisan cheese maker industries have applied selected bacterial strains as starting cultures in cheese ripening ([Supplementary-material supplementary-material-1]). In most cases, the starting cultures used managed to dominate cheeses' microbiome [22, 27, 30, 60, 61].

#### 2.3.2. Milk Microbiome

Crucial to the shaping of cheese microbiome communities is the microbial composition of milk. Analysis of Grana-like hard cheeses, a type of cheeses manufactured in the Piedmont region in northwest Italy, indicated that not only the starters existed in NWC but also the presence of contaminants in raw cow's milk, some of which were responsible for cheese spoilage, affected the microbiome profile in cheese, highlighting the importance of following proper hygiene practices in dairy farm environment, as well as during milking and milk processing [29]. A possible source of the milk microbiome, could be the animal's teat canal or the surface of teat skin [57]. Especially teat surface was found to contain increased amounts of coagulase negative staphylococci, members of the family *Enterobacteriaceae*, such as *Pseudomonas,* as well as coryneform bacteria and spoilage bacteria [62]. In another study, the existence of 85% common OTUs in raw milk and 27% in ripened cheeses with cow teat skin, further highlights the impact of farm's environment in shaping the microbiome developed in milk [61]. The animal used as a source of milk could also affect the profile of the microbial community formed in cheese. Cow's milk, was found to contain richer bacterial diversity compare to goat's and sheep's milk [51].

#### 2.3.3. Dairy Plant Environment and Equipment

Analysis of the effect of the dairy plant microenvironment, including work surfaces and tools used throughout dairies production procedure, on the microbial communities developed in dairy products, including ricotta, mozzarella, Caciocavallo, Grancacio, and Scamorza cheeses, in a dairy plant sited in the Campania region in Southern Italy, indicated that the species that are present in the dairy plant environment during processing may affect the shaping of the cheese microbiota [54]. This study highlighted also the importance of cleaning the surfaces and the tools used during cheese production process, to avoid the contamination of the product by spoilage bacteria. The dominant presence of *S. thermophilus* in dairy plant environment, was detected after analysis of the microbiome of two industrial dairy plants in Italy, producing the traditional Caciotta and Caciocavallo Pugliese cheeses [63]. *S. thermophilus* was detected not only in cheeses' rind and core, but also in every equipment used during cheese production, such as brine tanks, knife surface, drain table and ripening room. The other starters, including *Lactobacillus delbrueckii* subsp. *lactis*, *Lactobacillus helveticus* and *Lactobacillus lactis*, were found in lower relative abundance, underlining the increased colonization capability of *S. thermophilus* originating from its potential to convert lactose to lactate rapidly [64]. Analysis of the surface microbial community of two artisanal cheese-making plants via high-throughput sequencing indicated that the most dominant microbes in the majority of surfaces, were *Debaryomyces* and *Lactococcus* [65], which are among the dominant genera detected in numerous cheeses. The wooden equipment used during traditional Sicilian cheeses manufacturing was found to be a rich source of LAB, able also to produce antibacterial compounds against known pathogens, such as *L. monocytogenes* [66].

### 2.4. Processing Factors That May Affect PDO Cheeses Microbial Communities' Formation

#### 2.4.1. Processing Temperature Determines the Abundance of Thermophilic and Psychotrophic Microbes

Temperature is a basic factor implicated in microbes' variability; therefore, heating conditions should be strictly followed during PDO cheeses making. During incubation of the whey at increased temperature (39–54°C), the curd is dominated by the thermophilic LAB communities. Other subdominant species, such as mesophilic LAB, could be also found, the occurrence of which depends on cooking temperature and cooling treatment, creating complex dynamic interactions that influence the sensorial characteristics of the final product. Milk pasteurization is an important process applied to limit contaminants contained in raw milk, gained from the environment. Pyrosequencing analysis in Herve (PDO) cheese revealed a clear separation among the microbial communities of raw (heart and rind) compared to pasteurized (heart and rind) milk cheeses [44]. Furthermore, differences in the ripening temperature may affect the metabolism and consequently the growth rate of starter cultures, with consequent alterations in the cheese microbiome communities [22].

#### 2.4.2. Degree of Ripening

The quality of the produced cheeses, especially regarding cheeses lacking pasteurization, is greatly influenced from the degree of ripening. During ripening, mesophilic and thermophilic LAB dominate the cheese microbiome, limiting the presence of potential spoiler and pathogenic bacteria. Indeed, investigation of the effect of the degree of ripening in the bacterial communities' formation of three traditional Croatian ewe's milk cheeses, Istrian, Krcki and Paski revealed that the ripening procedure consequences alterations in the relative abundance of the lactic acid producing bacterial populations [46]. Due to the absence of pasteurization in these cheeses, the relative abundance of some pathogens, including *E. coli/Shigella flexneri, Salmonella* spp.*Pseudomonas* spp*., Serratia* spp., was high at day 0, but was reduced after 90 days of ripening due to the inhibitory activity of the dominant presence of the lactic acid bacteria. Similarly, the prevalence of LAB, including *Lactococcus*, *Lactobacillus* and *Streptococcus,* after 80 days of ripening, was shown at the end of ripening of the Plaisentif cheese, limiting milk spoilage genera, such as *Acinetobacter* and *Enhydrobacter,* which were present in milk and curd [67]. Investigation of the active microbiota during the ripening of a Grana-like cheese revealed that as the ripening progressed, beyond the microbiome that originated from natural thermophilic whey cultures, including *L. helveticus* and *L. delbrueckii,* and raw milk, such as *L. helveticus,Acinetobacter baumannii,S. thermophilus,* *L. delbrueckii* and *Propionibacterium acnes,* some other species, including members of *L. casei* group, which were present in limited relative abundance before ripening, became dominant [29]. During ripening, *L. casei* group species are able to survive sugar amount limitations due to their ability to utilize amino acids and peptides, as well as products of SLAB metabolism, as alternative energy sources [68] and this increase in the relative abundance of members of *L. casei* group was probably consequence of their ability to utilize the resulting products of the proteolytic properties of *L. helveticus.* In low quality raw milk, *L. helveticus* and *L. delbrueckii* were unable to dominate over some contaminant species, such as *P. acnes,* which remained metabolically active after 10 months of ripening. A progressive increase in mesophilic and thermophilic lactobacilli which was combined with a progressive decrease in mesophilic and thermophilic streptococci was revealed during ripening of Caciocavallo cheese [69]. Similarly, as in the case of Grana-like cheese, ripening limited the presence of potential spoilers including *Pseudomonas* sp. and elevated the abundance of *L. helveticus* and *L. delbrueckii,* although *S. thermophilus* remained the dominant species throughout ripening. At the end of ripening, a switch from thermophilic to mesophilic bacterial species, with *L. casei* group to become the subdominant population, was occurred.

Investigation of the factors contributing to traditional Minas artisanal cheese rind and core microbial communities shaping, starting from the metagenomic analysis of the microbiome of raw milk and of endogenous starter culture, indicated that abiotic factors, such as geographical location, acidity and moisture, affected the microbial communities' formation within sixty days of ripening [70]. More specifically, the families Leuconostocaceae and Lactobacillaceae, whose relative abundance was increased throughout ripening, were associated with reduced moisture values, whereas the families Streptococacceae, Pseudomonadaceae and Moraxellaceae indicated increased relative abundance in lower acidity parameters. Additionally, specific manufacturing practices, including routinely manipulation of the wheels by hand, were responsible for the prevalent presence of *Staphylococcus* in cheese rind. The family Planococcaceae, which was among the dominant families throughout ripening, seemed to interact strongly with the family Leuconostocaceae on the cheese surface, and this interaction was associated with regional factors, indicating a possible microbial signature of that type of cheese.

#### 2.4.3. Salt, Herbs and Spices Addition

Brine salting is a common practice applied in cheese making procedure. The high concentrations of NaCl contained in brine (5–25%) increase the osmotic pressure, preventing the growth of pathogens and preserving cheese for longer periods [71]. Feta-style cheese, for instance, which has increased salt content, lacked genera which are sensitive to high salt content, such as *Leuconostoc* and *Pseudomonas* [51]. The lack of salt sensitive species along with the fact that the high salt concentration increases the permeability of nutrients from curd to brine, favor the growth of halophilic and halotolerant species [72]. The presence of certain marine, halophilic and halotolerant LAB, such as *Alkalibacterium* and *Marinilactibacillus*, has been reported in Cotija cheese [21]. Soft brine-processed cheeses were found to exhibit lower pH values than hard and semi-hard cheeses. The increased acidity is attributed to the fact that soft cheeses undergo easiest rennet and organic acid (mostly lactic acid) coagulation in combination with easiest organic acids, mostly lactic acid, diffusion from the curd [73]. The detection of some pathogens in brines via HTS analysis, however, indicates that some unwanted species are able to tolerate high salt content [73].

Apart from salting, the addition of other ingredients, such as herbs and spices, has a crucial influence on cheese microbiome formation. Some of these ingredients possess antimicrobial activities, inactivating or preventing the growth of spoilage and pathogenic microbes [74]. Additionally, they affect the relative abundance of the core microbiome. Cheeses with adjunct ingredients, for example, showed reduced relative abundance of *Lactococcus* species [51].

#### 2.4.4. Specific Cheese Processing Conditions

16s rRNA sequencing analysis on the effect of the different manufacturing conditions on the traditional Polish, PDO, scalded-smoked cheese Oscypek, in curd, fresh and smoked samples, cheese microbiome identified that the most dominant genus in all samples was *Lactococcus*. The abundance of the latter was reported to increase after the smoking process, in contrast to the other dominant bacteria, *Streptococcus* and *Leuconostoc*, which were decreased [24]. The smoking process also decreased enterobacteria, indicating the positive impact of this procedure on the quality and safety of smoked traditional products. Different methods of curd acidification such as the addition of citric acid, or thermophilic defined or undefined starters supplementation, was found to affect the cheese microbiome composition of the high-moisture cow's milk Mozzarella cheese, affecting mostly the starters and the psychrotrophic spoilage organisms [60].

#### 2.4.5. Environmental Conditions

Lower bacterial diversity was observed during the dry, compare to the rainy season in analyzed Poro cheese samples [25]. Also, raw milk cheeses manufactured late during the day was found to have increased bacterial diversity compare to the cheese made earlier [75]. Cotija cheese distinctive flavors is believed to be affected from the environmental conditions, since ripening takes place in an open environment, affected by rain, humidity, and temperature, in combination with the fact that the cows graze freely due to the rich forest vegetation [21]. These factors influence the composition of the microbiome and consequently the flavor compounds produced due to the microbial metabolism.

### 2.5. HTS Technologies for Identifying the Influence of Microbial Metabolites to Cheese Ripening and Its Sensorial Characteristics

Fermented cheeses comprise a matrix rich in aromatic compounds, including aldehydes, alcohols, acids, esters and several ketones, lactones, pyrazines, sulfurous compounds, free fatty acids (acetic and propionic mainly), free amino acids, and salts produced from the microbial metabolism of lactose, lactate, fatty acids, sugars and proteins [33]. Flavor compounds are produced as a result of enzymatic activities from rennet, the starters and the other bacteria and yeasts, in combination with nonenzymatic reactions, affected by cooking temperature, ripening time, and fermentation/ripening temperature [11, 23, 76]. The degree of ripening and the manufacturing conditions affect the microbial communities formation and consequently their ability to utilize available carbon and nitrogen sources (amino acids, carbohydrates, carboxylic acids, amines, and polymers), as well as the degree of glycolysis (from lactose), lipolysis (from fat) and proteolysis (from caseins), leading to the production of different aromatic compounds [22]. Initially, the primary proteolysis of casein and fermentation of milk sugars is conducted by starter cultures, resulting in fast curd acidification and release of peptides and amino acids, and the secondary by nonstarter lactic acid bacteria (NSLAB) and secondary starters [77], resulting in the release of free amino acids (FAA) and branched compounds that enrich by then the cheese with aromatic and flavorful compounds.

Combination of metagenomic and metatranscriptomic analysis, may allow the identification of the most metabolically active microbes and their expressed genes with essential contribution in the process of cheese ripening [22, 77]. Analyzing the microbes in surface-ripened cheeses indicated that at the initiation of ripening, enzymes responsible for the fermentation of lactose were found to be expressed by *L.lactis* and *Kluyveromyces lactis,* whereas for lactate degradation some yeasts, including *Debaryomyces hansenii,G*. *candidum* and *Saccharomyces cerevisiae* were reported to be the most likely candidates [78]. Additionally, a large number of transcripts indicating the proteolytic and lipolytic action of *L*. *lactis* were identified, even though the basic contributor to proteolysis and lipolysis throughout ripening was found to be *G*. *candidum.* Similarly, investigation of the alterations in transcription level throughout the ripening process of Reblochon-type cheeses, in which *Streptococcus thermophilus* and *Lactobacillus bulgaricus* (SLAB), *Brevibacterium aurantiacum* (ripening bacterium), *Debaryomyces hansenii* and *Geotrichum candidum* (yeasts) were applied before ripening, in combination with biochemical analysis, indicated that after the catabolism of casein and lactose by LAB, a switch from the metabolism of galactose and lactate to amino acids catabolism was observed throughout ripening [79]. This shift was combined with decrease in the abundances of transcripts involved in galactose catabolism and ammonia assimilation, and increase in transcripts involved in the degradation of amino acids such as glutamate, proline, glycine, serine and threonine, in the yeasts *G. candidum* and *D. hansenii*.

Analysis of the contribution of the most abundant species based on DNA-seq and RNA-seq analysis, in Maasdam cheese sensorial characteristics revealed the ability of *L. lactis*, *L. rhamnosus*, and *Propionibacterium freudenreichii* to produce the buttery flavor compounds R-acetoin and diacetyl and *L. lactis* to produce 2,3-butanediol [22]. *L.lactis* and *P. freudenreichii* contained genes for valine degradation, associated with malty and fruity flavors, while *L. lactis, L. rhamnosus,* and *L. helveticus* were able to produce volatiles such as methanethiol and hydrogen sulfide, from sulfuric amino acids degradation. Metagenomic analysis, in combination with metagenomic assembly of the genomes of the three most abundant species, *Lactobacillus plantarum, Leuconostoc mesenteroides* and *Weissella paramesenteroides*, in Cotija cheese's microbiome, identified the presence of genes involved in the production of α-keto acids, aldehydes, ketones, alcohols, esters or thioesters, thiols, carboxylates and benzene derivatives, e.g. toluene and xylene [21]. *De novo* assembly of the genomes of the dominant fungus *Geotrichum candidum* and *Penicillium camemberti* in a commercial Canadian Camembert-type cheese identified transcripts corresponding to energy metabolism procedures, including glycolysis/gluconeogenesis, pentose phosphate pathways, tricarboxylic acid cycle, oxidative phosphorylation and the glyoxylate bypass [80]. Lyases implicated in volatile sulfur compounds (VSC) formation via methionine catabolism as well as transcripts related to cabbage, sulfur aroma and ammonia production from methanethiol, methylketones and secondary alcohols production, were also found.

Levante et al. [81] performed a combinatory 16S rRNA HTS analysis with spxB HTS analysis and pyruvate oxidase pathway activation analysis via reverse transcription real time PCR (RT-qPCR), to distinguish the metabolically active bacteria throughout the long ripened Grana Padano PDO cheese ripening, as well as to identify the NSLAB species that contribute to its sensorial characteristics. The SpxB gene which codes for pyruvate oxidase, allows the discrimination of the members of the *L. casei* group. These analysis led to the identification of four clusters inside the dominant throughout ripening *L. casei* group. Based on that, analysis of additional metabolic genes via HTS may provide insights for the metabolically active and the metabolic behavior of the bacterial strains associated with cheese ripening.

### 2.6. Combination of HTS Methodologies with Metabolomics Offers a Novel Way to Characterize the Effect of Microbial Communities to PDO Cheeses Sensorial Characteristics

The identification of the dynamic contribution of the microbial community, as well as the individual contribution of members of the community to sensorial characteristics development includes the combination of HTS with metabolomics. Association study of the microbial spatial distribution with secondary proteolysis as well as with volatile organic compounds (VOC) produced, performed in Pecorino Siciliano, Pecorino Toscano and Fiore Sardo cheeses, revealed correlation among mesophilic lactobacilli (mostly *L*. *plantarum*) with numerous VOCs, including aldehydes, esters, sulfur compounds, alcohols and FAA with thermophilic lactic acid bacteria (including *L*. *delbrueckii and S*. *thermophilus*), as well as some halophilic, aerobic, or aero-tolerant bacteria with VOCs such as esters, ketones, aldehydes, furans and sulfur compounds [82]. In a similar study, *Lactobacillus* spp. and *L. paracasei* were associated with the concentration of total FAA and the area of hydrophilic peptide peaks and mostly *L. paracasei* with the amino acids Glu, Asp, Leu, Val, and Phe, as well as with several aldehydes, alcohols, ketones, esters, sulfur compounds and all furans. *St. thermophilus* was associated with carbohydrates and amines utilization as well as with the VOC acetaldehyde, 2,3-butanedione (diacetyl), and 2,3-pentanedione production [83].

Analysis of the microbial population dynamics and the flavor production dynamics throughout ripening of the fermented Kazak artisanal cheese, with combination of NGS, reversed phase high performance liquid chromatography (HPLC) and gas chromatography/mass spectrometry (GC/MS), revealed strong correlation between some dominant genera, including *Lactococcus,Lactobacillus*, *Bacillus,Acetobacter*, *Staphylococcus*, *Moraxella andKurthia* regarding bacteria, *Kluyveromyces*, *Aspergillus*, *Issatchenkia* and *Candida* regarding fungus, and the producing amino acids and fatty acids, as well as some volatile components [84]. Similarly, associations among the amino acids glutamic acid (Glu), isoleucine (Ile), histidine (His), and proline (Pro) with the genera *Lactococcus, Bacillus, Acetobacter, Staphylococcus, Moraxella* and *Kurthia*, the fungus *Dipodascus* with 9-octadecenoic acid, *Pichia* and *Penicillium* with (Z)-9-hexadecenoic acid, and *Candida* as well as *Issatchenkia* with the amounts of phenylalanine (Phe) and leucine (Leu) [85]. Other microorganisms, including *D. hansenii,Glutamicibacter arilaitensis,* as well as *Geotrichum candidum* and *Brevibacterium linens* were associated with the synthesis of carboxylic acids and alcohols, with carboxylic acids, alcohols and ketones, and with sulfur compounds respectively [86].

The influence of the metabolic capabilities of starter lactic acid bacteria (SLAB), including *Lactococcus lactis, Lactobacillus delbrueckii* and *Streptococcus thermophilus*, as well as NSLAB on volatile organic compounds (VOCs) including flavors production was indicated recently using gas chromatography mass spectrometry (GC-MS) [87]. Evaluation of the viability of *L. lactis* subsp*. lactis* starter cultures during 180 days of ripening and their ability to produce aroma compounds, as analyzed by Gas Chromatography-Mass Spectrometry (GC-MS) and estimated by correlation with the expression of *metC* and *als* genes, indicated viability of *L. lactis* subsp*. lactis* throughout cheesemaking and ripening and association with the cheese flavors acetoin, diacetyl, 2,3-butanediol and dimethyl disulfide [88]. The synergistic metabolic activity of *Lactobacillus bulgaricus* and *S. thermophilus* was shown to improve the fermentation process and the stability of the cheese produced, as well as to stimulate the production of exopolysaccharide and aromatic compounds [89].

### 2.7. Combination of HTS Methodologies with Metaproteomics to Ensure the Quality of PDO Cheeses

In order to sustain and/or increase their economic income influenced by the good reputation of PDO cheeses, the producers require to guarantee the good quality and safety for the consumers of their products, especially regarding PDO cheeses that luck pasteurization. The early detection of the presence of particular spoilage microbes may prevent their detrimental consequences for the cheese value. Metaproteomic approach was applied to estimate the contribution of bacterial metabolism on Grana Padano cheese quality [90]. The conduction of butyric fermentation by some Clostridia develop in Grana Padano cheese due to contamination, spoils the quality of the product, causing late blowing. Although NGS was not applied, metaproteomics revealed some enzymes that altered due to the application of lysozyme treatment. If the researchers had combined metaproteomics with metagenomic applications, the dynamics of the presence of specific microbes associated with particular metabolic pathway responsible for spoilage would allow the better evaluation of the contribution of that microbes as members of cheese microbiome to cheese quality.

### 2.8. Additional Advantages and Limitations of Technological Uses of HTS in Dairy Industry

#### 2.8.1. Advantages

The significant benefits in the cheesemaking process *via* the use of HTS analyses are gradually arising. NGS technologies have significantly enhanced the capability to recognize and characterize the cheese microbiome and its metabolic and monitoring capacities through which the microbes could affect the sensorial characteristics of the final product. The identification of the characteristic microbial consortium comprising cheeses was revealed to be important for understanding not only the contribution of the added started cultures, but the other microbes in the final sensorial characteristics. Evaluation of the effects of varying the ripening temperature of traditional Italian cheese, lead to the identification of the critical contribution of NSLAB in the ripening process [23]. Specifically, elevated ripening temperature increased cheese maturation rate regarding the degree of lipolysis, proteolysis and lipid/amino acid catabolism. Furthermore, this analysis may reveal potential biomarkers for assessment of the normal progression of the cheese ripening process and aroma compounds production. The expression levels of phosphoenolpyruvate kinase (PEPCK), an enzyme essential for the gluconeogenesis pathway of sugars from lactate, was proposed to be suitable biomarker for estimating the ordinary progression of the Camembert-type cheese ripening procedure [80]. Since an increasing body of research accumulates more HTS generated data of higher quality and from a broader spectrum of sources, will lead to better understanding of the microbial physiology during the different levels of the ripening procedure. Consequently, this will allow us to decipher the formation of unique sensorial characteristics, improve the manufacturing to ensure safety, authenticate and protect the origin of various cheeses.

#### 2.8.2. Limitations

Although not in the scope of the current review, some limitations include the elevated cost of genome and transcriptome sequencing and the errors elicited during reading the data [43]. Additionally, since the currently available platforms can only detect with accuracy taxa reaching the genus level, the detection of some food-borne pathogens, or other metabolically active species, demands extra, strain specific and further sensitive methods, including qPCR and ELISA [91, 92], or shotgun sequencing to be combined with cultured based methods.

Other limitations of HTS include fluctuations in measurements due to sample processing, DNA isolation and the sequencing process. For example, Gram-negative bacteria cell wall is degraded more easily compared to gram-positive bacteria and partial lysis might indicate false relative bacterial representation [93]. Additionally, the extraction potential of each kit or each protocol followed, varies in quantity and quality of the extracted microbial DNA [51, 94]. During the process of library preparation, the PCR-reaction can only amplify a fragment of the 16S or 18S rRNA gene, ranging from 100 to 600 bases, to be sequenced with the currently HTS available platforms and each sequencing platform applies different primers targeting different conserved regions [95, 96]. Another important factor to be considered is the next-generation sequencing platform used. The currently available sequencing platforms include Illumina MiSeq and HiSeq, Roche 454, and Thermo Fisher Ion Torrent, which use different DNA sequencing chemistries to perform the DNA sequencing, and apply different approaches for library preparation, strategy of sequencing as well as the tools used for bioinformatic analysis [42]. Differences in the amplicon sequencing procedures, in the concentration of the PCR template, in the conditions of the amplification, problems extracted from pooling multiple barcodes and other variations extracted from the sequencing itself have consequent alterations in the sequenced amplicon size, the number of reads produced and the sequencing accuracy [97]. The depth of sequencing can reveal microbes existing in very low relative abundances, that otherwise would be disregarded [98]. Furthermore, the massive production of enormous data sets elicited from sequencing are difficult to analyze and require sophisticated computational systems in combination with bioinformatic tools [99].

### 2.9. Machine Learning Approaches to Study the PDO Cheeses Microbiome

HTS technologies generate a large amount of data that often require extensive statistical and bioinformatics approaches for analyzing them, enabling the precise classification of different types of cheeses. In conjunction with HTS technological advancements to provide larger datasets with minimal error in the measurements, these bioinformatics approaches are becoming an indispensable part of HTS workflows. Consequently, any application of HTS in supporting cheese PDO will have to undergo through a robust and precise analytics pipeline.

Identification and quantification of biases in the microbial ecology require multivariate analytics and machine learning approaches for pattern identification, clustering and classification [100, 101]. The potential utility of these approaches is evident in an increasing body of literature on cheese metagenomics. Li et al. [102] by applying Principal Coordinate Analysis (PCoA), hierarchical clustering and have identified the distinct patterns while using Redundancy Analysis (RDA), have explained a large percentage of the observed variability across the highly abundant genera between cheeses produced in different geographical locations (i.e. Kazakhastan, Italy, Belgium and Kalmykia). Permutation analysis was utilized by Calasso et al. [63] to characterize the catabolic profiles of microbes found in dairy products, including milk and cheeses and on their preparation equipment while Matera et al. [103] discuss the variability in the volatile compounds in cheese via Principal Component Analysis (PCA). More recently, Linear Discriminant Analysis (LDA) was employed in LEfSe, an algorithm for high-dimensional biomarker discovery [104]. LEfSe initially identifies statistical significant features (e.g. differentially abundant OTUs) that exhibit differences across classes of interest followed by testing of their biological consistency using non-parametric statistical analysis. Finally, it estimates the effect size of each differentially abundant feature of the dataset. LEfSe was utilized in analyzing metagenomic data from 137 rind microbial communities extracted from cheeses across 10 countries [48].

While several studies have extensively characterized microbial communities in cheeses using the methods described above, their majority was limited to profiling based on phylogenetic gene markers like 16S rRNA. Consequently, there is a need of functional characterization for gaining insights into the microbial processes in ripening cheese. However, whole metagenome sequencing for determining the gene content of microbial samples is prohibitively expensive, therefore computational predictions are essential for addressing this challenge. PICRUSt (Phylogenetic Investigation of Communities by Reconstruction of Unobserved States) is a recently proposed method designed specifically to address this challenge [105]. By integrating the phylogenetic gene markers obtained from 16s with a reference phylogenetic tree, PICRUSt can infer the gene content and its expected abundance of gene families in the microbial community under study. This approach was validated using over 500 metagenomics samples from the Human Microbiome Project (HMP) [106] and has been successfully applied in predicting the functional composition in terms of gene families in Iranian Liqvan cheese [107].

**Figure 2:**
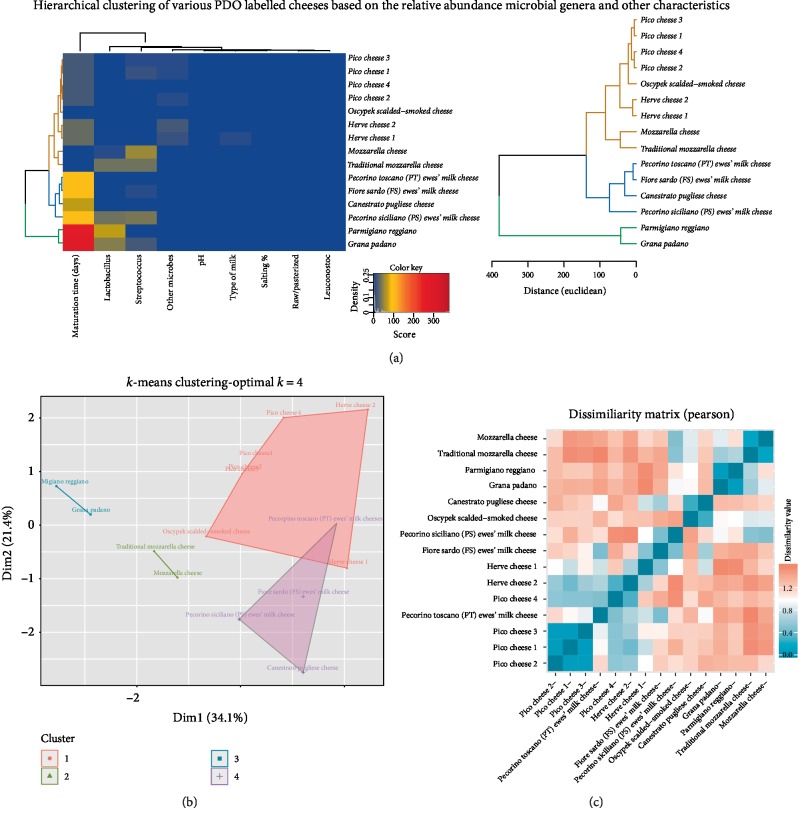
The figure shows the result of Hierarchical (a) *k-*means, (b) clustering and the dissimilarity matrix, (c) between all pairwise combinations of the cheeses shown in Supplemental [Supplementary-material supplementary-material-1]. All methods identify a large cluster of cheese that are characterized by high relative abundance of *Lactococcus*. High relative abundance of *Streptococcus,* Lactobacillus or other genera are characteristic for smaller clusters.

While the demonstration of a thorough analytics workflow is beyond the scope of this review, Figure 1 shows an example of applying clustering algorithms on a limited metagenomics dataset. The latter was derived from the reported relative abundances of microbial organisms in the literature which is discussed in this review [24, 26, 28, 30, 44, 45]. Here, considering the 4 most represented genera i.e. *Lactococus*, *Lactobacillus*, *Streptococcus* and *Leuconostoc* across the cheeses shown in Supplemental [Supplementary-material supplementary-material-1], we have applied hierarchical clustering, k-means and dissimilarity Pearson metric algorithms in R [108].  Figure 2(a) shows the hierarchical clustering results based on the Euclidean distance between of each cheese in the 5 dimensions (4 genera and 1 other genera). The largest cluster is characterized by high abundance in *Lactococcus* and includes mainly soft to semi-hard cheeses. Smaller clusters are characterized with high abundance of *Streptococcus* and/or *Lactobacillus*, high levels of *Lactobacillus* only (in particular the cheeses inoculated with NWC) and finally a cluster where other genera are dominant. Similar clusters are shown with the k-means (Figure 2(b)) and dissimilarity metric (Figure 2(c)) albeit with some differences originating from the limitations of each method. In conclusion, the results above reinforce the realization that cheeses of the same type produced from different farms (e.g. Pico 1-4 or Paski 1 & 2 cheeses) and cheeses that undergo similar fermentation/ripening processes fall within the same clusters as they share similar microbial profiles.

## 3. Conclusion

Each type of fermented cheese is comprised by a unique microbiome shaped by the influence of several factors reflecting regional differences along with differences in the specific production process. The fermentation processes that take place during cheese ripening influence the development of distinct sensorial characteristics. HTS methodologies have greatly increased our knowledge regarding cheeses' microbial diversity and have revealed some impacts of the microbial metabolism on sensorial characteristics formation. The recognition of the role of specific microbes in the fermentation procedure, the identification of the factors shaping the cheeses' microbiome both regarding their diversity, abundance but also the functional and metabolic processes, constitute the unique signature of each cheese type and are essential factors for establishing the quality of fermented cheeses, although more effort needs to be provided to understand the contribution of each member of the community to cheese sensorial characteristic shaping. Identifying this microbial signature through HTS technologies can be a powerful tool for supporting PDO and other authentication accreditations. Whilst some limitations exist in substituting the traditionally used methodology for PDO establishment, HTS technologies as they become faster, more accurate and cost effective can complement and expedite this process.

## Figures and Tables

**Table 1:** Advantages and limitations of technological uses of HTS in dairy industry.

Advantages and limitations of technological uses of HTS in dairy industry	
Advantages	Limitations
(i) Identification and characterization of cheese microbiome.	(i) Elevated cost of genome and transcriptome sequencing.
(ii) Understanding how microbial metabolic and monitoring capacities may affect cheese sensorial characteristics.	(ii) Errors elicited during reading the data.
(iii) Evaluation of the effects of cheese manufacturing conditions in cheese microbial communities and sensorial characteristics development.	(iii) The currently available platforms can only detect with accuracy taxa reaching the genus level, so the detection of some food-borne pathogens demands additional methods.
(iv) Identification of potential biomarkers for evaluation of normal cheese ripening process and aroma compounds production.	(iv) Fluctuations in measurements due to sample processing, DNA isolation and the sequencing process.
(v) Better understanding of the microbial physiology during the different levels of the ripening procedure.	(v) Require sophisticated computational systems in combination with bioinformatic tools.
(vi) Allow the improvement of cheese manufacturing to ensure safety, authenticate and protect the origin of various cheeses.	

## Supplementary Materials

Supplementary Materials Supplementary Table 1 provides information about several PDO and other traditional cheeses discussed in the manuscript regarding the type of cheese, the milk source, their origin and the addition of starter cultures. Additionally, the table provides information for the methods of chesses' analysis, including the DNA/RNA extraction method, the primers and the region of their target gene and the sequencing platform used. Finally, the results of the metagenomics analysis as relative abundances of bacterial and/or fungal genera/species and the extracted from the analysis number of sequences are also shown.
