# Supplementary material for: High Throughput Sequencing Technologies as a New Toolbox for Deep Analysis, Characterization and Potentially Authentication of Protection Designation of Origin Cheeses?
Source: Int J Food Sci. 2019 Nov 20;2019:5837301. doi: 10.1155/2019/5837301 (PMC6925717; doi:10.1155/2019/5837301)
Supplement: Supplementary Materials — Supplementary Table 1 provides information about several PDO and other traditional cheeses discussed in the manuscript regarding the type of cheese, the milk source, their origin and the addition of starter cultures. Additionally, the table provides information for the methods of chesses' analysis, including the DNA/RNA extraction method, the primers and the region of their target gene and the sequencing platform used. Finally, the results of the metagenomics analysis as relative abundances of bacterial and/or fungal genera/species and the extracted from the analysis number of sequences are also shown. [file 5837301.f1.pdf]

Supplementary table 1 provides information about several PDO and other traditional cheeses discussed in the manuscript regarding the type of cheese, the milk source, their origin and the addition of starter cultures. Additionally, the table provides information for the methods of chesses' analysis, including the DNA/RNA extraction method, the primers and the region of their target gene and the sequencing platform used. Finally, the results of the metagenomics analysis as relative abundances of bacterial and/or fungal genera/species and the extracted from the analysis number of sequences are also shown.

Table S1. Metagenomic studies on PDO and other traditional cheeses. Differences in the relative abundance of microbes (bacterial and fungal representatives) based on the cheese type, the source of milk, the origin and the methods of analysis.

Type of cheese	Milk source	Origin	Starter cultures	Extraction method	Target region - Primers	Bacterial representatives	Fungal representatives	Number of sequences	Reference
**Sequencing platform: 454 pyrosequencing system FLX platform**									
Istrian cheese	raw, sheep milk	Istria	no	Maxwell Tissue DNA Purification Kit (Promega, Madison, USA).	927f (5'-AAACTYAAAKGAATTG ACGG-3', *E. coli* position 908–927) and 630r (5'-CAKAAAGGAGGTGATC C-3', *E. coli* position 1529 to 1545)	**Farm 1**. *L. lactis*: 86.47%, *Enterococcus spp*. 7.05%. **Farm 2.** *L. lactis*: 3.89%, *Enterococcus spp*. 42.65%, *L. mesenteroides*: 1.39%, *S. parauberis*: 40.33%, *S. saprophyticus*: 1.14%, *E.coli/Shigella flexneri*: 3.14%	Not analyzed	50544 high-quality partial 16S rRNA gene sequences, in average 2777 sequences per treatment	(Fuka *et al.* 2013)
Krcki cheese	raw, sheep milk	Istria	no			**Farm 1**. *L. lactis*: 99.22%. **Farm 2.** *L. lactis:* 83.37%, *Pediococcus pentosaceus*: 2.10%, *L. acidipiscis*: 2.96%, *S.gallolyticus*: 5.87%, *S. saprophyticus*: 2.67%	Not analyzed		
Paski cheese	raw, sheep milk	Istria	no			**Farm 1.** *L. lactis*: 11.37%, *Enterococcus spp*. 64.60%, *L. mesenteroides*: 14.48%, *S. gallolyticus*: 1.22%, *E.coli/Shigella flexneri*: 0.34%. **Farm 2.** *L. lactis:* 36.75%, *Enterococcus spp*. 48.22%, *L. mesenteroides*: 1.46%, *S. parauberis:* 2.59%	Not analyzed		
Poro cheese	Holstein cow milk	river zone of Tabasco, Mexico	fermented whey	phenol-chloroform DNA isolation	V1–V3 region: 27Fmod and 530R	*>90% Streptococcus* and *Lactobacillus.* Most representative species: *S. salivarius* subsp. *thermophilus* and *L. delbrueckii*. Occasionally: *Staphylococcus*, *Acinetobacter*, *Chryseobacter*, *Bacillus*, *Sediminibacterium*, *Enterococcus* and *Lactococcus*	Not analyzed	693,746 reads. Total OTUs: 273 97% similarity	(Aldrete-Tapia *et al.* 2014)
Fiore Sardo (FS) ewes' milk cheese	raw ewes' milk &	Sepi Formaggi, Italy	lamb rennet paste	RiboPureTM—Bacteria Kit (Ambion RNA, Life Technologies Co., Carlsbad, CA, USA)	V1–V3 region (*Escherichia coli* position 27–519, forward 28F and reverse 519R	*Lactococcus*: 82.4 to 91.8%, *Streptococcus:* 7.8 to 14.9% and *Lactobacillus*: 0.4 to 2.6%. *L. lactis:* 82.4 to 91.8%, *S. thermophilus:* 7.8 to 14.9%, *Lactobacillus* sp.: 0.3 to 2.1%.	Not analyzed	FS: 50,290 PS: 34,932 PT: 57,640 ESC: >99%. 29, 51 and 29 OUT respectively.	(De Pasquale *et al.* 2016)
Pecorino Siciliano (PS) ewes' milk cheese	raw ewes' milk	Casa del Formaggio Sant'Anna, Italy	lamb rennet paste			*Lactobacillus*: 26.0 to 66.3%, *Streptococcus*: 32.7 to 73.8%, *Enterococcus:* 0.1 to 0.7%, *Staphylococcus:* 0.1 to 2.4%, *S. thermophilus:* 44.5 to 72.3%, *L. delbrueckii:* 7.2 to 10.9%, *Lactobacillus* sp.: 51.3 to 58.8%.	Not analyzed		
Pecorino Toscano (PT)	pasteurized	Spadi Enzo, Italy	*Lactococcus lactis* -			*Lactococcus:* 90.9 to 99.9%, *Brachybacterium:* 0.02 to 0.9%, *Arthrobacter:* 0.05 to 0.2%, *Brevibacterium:* 0.1 to 4.4%, *Halomonas* 0.1 to 2.6%. *Lc. lactis:* 90.9 to 99.9%.	Not analyzed		

ewes' milk cheese	ewes' milk		calf rennet paste						
Caciocavallo Pugliese cheese	raw cows' milk	Noci, Bari (Apulia), Italy.	*Streptococcus thermophilus*	RiboPure bacterial kit (Ambion RNA; USA)	V1-V3 region (*E. coli* positions 27 to 519; 28F and 519R) of the 16S rRNA	90th day: *Lactobacillus* sp. 2.13% *Lactobacillus casei:* 3.28%, *Lactobacillus paracasei:* 10.86%, *Streptococcus* sp.: 16.60%, *Streptococcus thermophilus:* 66.42%	Not analyzed	197,385 raw sequence reads ESC >99%	(De Pasquale *et al.* 2014)
Pico cheese	Raw milk	Azores (Portugal)	animal rennet	Invisorb Spin Tissue mini-kit	e V3 and V4 region, forward primer 5'-ACTCCTACGGGAGGCAG-3' and reverse primer 5'-TACNVRRGTHTCTAATYC-3'	*Lactococcus*: 77.734%, *Staphylococcus*: 1.329%, *Exiguobacterium*: 1.042%, *Acinetobacter*: 7.316%	Not analyzed	65,746, 72,592 and 63,371 from cheese-makers A, B and C respectively ESC >99%	(Riquelme *et al.* 2015)
	Raw milk		animal rennet			*Lactococcus*: 75.032%, *Streptococcus*: 13.197%, *Enterococcus*: 4.047%, *Macrococcus*: 1.079%, *Acinetobacter*: 2.85%	Not analyzed		
	Raw milk		animal rennet			*Lactococcus*: 78.811%, *Streptococcus*: 2.716%, *Lactobacillus*: 1.39%, *Acinetobacter*: 2.47%, *Marinomonas*: 1.191%, *Pseudomonas*: 1.447%	Not analyzed		
	pasteurized cow milk		*DELVO®TEC DX-33, DSM, The Netherlands*			*Lactococcus*: 77.119%, *Streptococcus*: 9.119%, *Enterococcus*: 9.004%, *Lactobacillus*: 2.990%	Not analyzed		
Oscypek scalded-smoked cheese	raw sheep's milk	Tatra Mountains Poland	No	Genomic Mini DNA purification kit	V5 and V6 regions of the 16S rRNA gene	**Curd:** *Leuconostoc*: 3.121%, *Lactococcus*: 64.936%, *Streptococcus*: 6.356%. **Fresh:** Lactobacillus: 1.522%, Leuconostoc: 2.355, Lactococcus: 68.846%, Streptococcus: 5.181%. **Smoked:** Lactobacillus: 1.68%, Leuconostoc: 2.115, Lactococcus: 75.473%, Streptococcus: 4.130%.	Not analyzed	Curd: 5.179 Fresh cheese: 59.451 Smoked cheese 46,894 ESC 99%	(Alegría *et al.* 2012)
**Sequencing platform: 454 pyrosequencing on a GS Junior platform (454 Life Sciences, Roche Diagnostics, Italy)**									
Traditional mozzarella cheese	whole raw water buffalo's milk	southern Italy, provinces of Salerno (M1) and Caserta (M2)	NWC1: *S.thermophilus, L.delbrueckii, L.helveticus.* NWC2: *S.thermophilus, L.delbrueckii*	Biostic bacteremia DNA isolation kit (Mo Bio Laboratories, Inc., Carlsbad, CA).	V1-V3 region of the 16S rRNA gene by using the primers Gray28F (5'-TTTGATCNTGGCTCAG) and Gray519r (5'-GTNTTACNGCGGCKGCTG)	***M1:*** *L.delbrueckii*: 8%, *L. helveticus*: 20%, *L.kefiranofaciens: 10%, Lactobacillus sp.: 10%, S. thermophilus*: 45%. **M2:** *L. delbrueckii*: 48%, *L.lactis:* 3%, *S.thermophilus*: 40%.	Not analyzed	10,219 for M1 and 5,056 for M2. ESC >93.6%	(Ercolini *et al.* 2012)
PDO Canestrato Pugliese cheese	raw ewes' milk	Biccari, Foggia, Apulia region, Italy.	-	RiboPure-Bacteria kit	V1-V3 region	90 days of ripening: *Lactococcus*: 87.2% (*L. lactis, L. garvieae* & *L. raffinolactis) Lactobacillus* (4.8%) & *Leuconostoc* (3.9%).	Not analyzed	66,924 raw sequence reads ESC >99%	(De Pasquale *et al.* 2014)
hard-pressed Grana-type cheese	Frisona cow raw milk	Piedmont region, northwest Italy	whey starter	(Rantsiou *et al.* 2008) & Master-Pure complete DNA and RNA purification kit	V1 to V3 region	D batch: *L. casei*: 37%, *L. brevis*: 6.8% and *S. thermophilus*: 10.8% F batch: *P. acnes*: 42%, *Staphylococcus:* 5%. Batch E: *L. helveticus, L. casei* & *L. delbrueckii* ~70%, *S. thermophilus, P. acnes* ~20%.	Not analyzed	148,944 reads passed filters, average: 3,819 reads/sample ESC >99%	(Alessandria *et al.* 2016)

Ricotta cheese	-	Campa nia region (South ern Italy) provinc e of Salerno	whey starter	Biostic Bacteremia DNA isolation kit (Mo Bio Laboratories, Inc., Carlsbad, CA).	V1-to-V3 region of the 16S rRNA gene D1-D2 domain of the 26S rRNA gene, using the primers NL-1 (5’-GCATATCAATAAGCGG AGGAAAAG-3’) and NL-4 (5’-GGTCCGTGTTTCAAGA CGG-3’)	*Chryseobacterium*: 2%, *Chryseobacterium bovis*: 1%, *Wautersiella*: 1%, *Sphingobacterium faecium*: 4%, *Exiguobacterium*: 1%, *Lactobacillus zeae*: 1%, *Kurthiagibsonii*: 1%, *S. thermophilus*: 25%, *Agrobacterium*: 1%, *Comamonas*: 2%, *Delftia*: 1%, *Aeromonas*: 3%, *Acinetobacter*: 2%, *Acinetobacter johnsonii*: 4%, *Acinetobacter*: 1%, *Pseudomonas*: 30%, *Shewanella*: 8%, *Vibriorum oiensis*: 1%, *Stenotrophomonas*: 2%	*Penicillum solitum*: 19% *Debaryomyces hansenii*: 28%, *Yamadazyma triangularis*: 46% *Candida pararugosa*: 4%, *Cryptococcus curvatus*: 1%, *Trichosporon faecale*: 2%	A total of 117,490 reads passed the filters, ESC 99%	(Stellat o *et al.* 2015)
mozzarel la cheese	-		natural whey cultures			*Chryseobacterium*: 1%, *Leuconostoc*: 13%, *Lactococcus*: 1%, *Streptococcus thermophilus*: 75%, *Pseudomonas*: 5%, *Shewanella*: 1%	*P. solitum*: 6%, *D. hansenii*: 19%, *Y.triangularis*: 52%, *Dipodascus capitatus*: 2%, *Galactomyces geotrichum*: 5%, *Geotrichum klebahnii*: 1%, *Candida pararugosa*: 3%, *C. zeylanoides*: 1%, *Kluyveromyces marxianus*: 1%, *Rhodotorula glutinis*: 1%, *C. curvatus*: 1%, *T.faecale*: 5%, *T. lactis*: 1%, *T.quehoae*: 1%		
Caciocav allo cheese	-		natural whey cultures			*Lactobacillus*: 4%, *Lactobacillus delbrueckii*: 6%, *Lactobacillus helveticus*: 2%, *Lactobacillus zeae*: 13%, *Streptococcus thermophilus*: 72%.	*P. solitum*: 1%, *D.hansenii*: 4%, *Y. triangularis*: 12%, *D.capitatus*: 11%, *G.geotrichum*: 4%, *Geotrichum fragrans*: 5%, *C. pararugosa*: 3%, *K. marxianus*: 41%, *Saccharomyces cerevisiae*: 8%, *C.curvatus*: 1%, *T. faecale*: 4%, *T. lactis*: 2%, *T.quehoae*: 1%		
Grancaci o cheese	-		natural whey cultures			*Lactobacillus*: 1%, *Lactobacillus delbrueckii*: 7%, *Lactobacillus zeae*: 14%, *Streptococcus thermophilus*: 68%, *Chromohalobacter Canadensis*: 2%, *Pseudomonas*: 4%, *Shewanella*: 1%	*P. solitum*: 3%, *D. hansenii*: 12%, *Y. triangularis*: 79%, *C. pararugosa*: 1%, *T.faecale*: 2%		
Scamorz a cheese	-		natural whey cultures			*Chryseobacterium*: 1%, *Lactobacillus helveticus*: 1%, *Leuconostoc*: 11%, *Lactococcus*: 1%, *Lactococcus lactis*: 1%, *Streptococcus thermophilus*: 71%, *Arcobacter*: 2%, *Aeromonas*: 1%, *Acinetobacter*: 1%, *Acinetobacter johnsonii*: 1%, *Pseudomonas*: 5%, *Shewanella*: 1%.	*P. solitum*: 8%, *D. hansenii*: 19%, *Y.triangularis*: 45%, *D.s capitatus*: 1%, *G. geotrichum*: 9%, *G.fragrans*: 1%, *G. klebahnii*: 1%, *C. pararugosa*: 2%, *K. marxianus*: 2%, *R. glutinis*: 1%, *C.curvatus*: 1%, *T. faecale*: 4%, *T. lactis*: 1%, *T. quehoae*: 4%		
Mozzare lla cheese	raw water buffalo’ s milk	Campa nia region, Southe rn Italy	natural whey cultures	Biostic bacteremia DNA isolation kit (Mo Bio Laboratories, Inc., Carlsbad, CA).	V1–V3 region of the 16S rRNA	*S. thermophilus* 70%, *Lactococcus lactis* 13%, *Lb. fermentum* 12%	Not analyzed	221,903 reads passed the filters, average value: 4,191 reads/sample ESC >99%	(De Filippis *et al.* 2014)
Grana Padano	Raw cow’s milk	Grana Padano Northe rn Italy	natural whey cultures			*Lb. delbrueckii* & *Lb. helveticus* 59%, *S. thermophilus* >24%,	Not analyzed		

Parmigiano Reggiano	raw, cow's milk	Parmigiano Reggiano, Northern Italy	natural whey cultures			*Lb. delbrueckii* & *Lb. helveticus* were 93%, *S. thermophilus* in lower abundance	Not analyzed		
Fontina (PDO) cheese	raw cow's milk	Northwest Italy (Aosta Valley)	*S.thermophilus*, *L.delbrueckii* and *L.lactis*	(Rantsiou *et al*. 2008)	V1–V3 region of the 16S rRNA gene by using primers Gray28f & Gray519r	*S. thermophilus* & *L. delbrueckii, L. casei, L. lactis in some samples* with a very low incidence, *Enterobacteriaceae*, *Pseudomonas* and *P. acnes* were detected with moderate incidence	Not analyzed	134,171 reads passed filters, average: 4969 reads/sample ESC > 97%	(Dolci *et al.* 2014)
High-moisture Mozzarella cheese	cow's milk	Italian market	*S.thermophilus*, *L. delbrueckii*, *L. helveticus* *L.lactis*, *Leuconostoc*, *Enterococcus*	MoBio Power food Bacterial DNA Extraction kit (MO BIO Laboratories Inc., USA)	V1–V3 region of the 16S rRNA gene Primers Gray28F & Gray519r	Cheeses produced by direct citric acid addition: starter microorganisms: 6 to 40%. Pshychrotrophic taxa and *Pseudomonas:* highest median frequency. Cheeses produced by addition of defined starter cultures: *S. thermophilus* the dominant microorganisms and the lowest contamination with psychrotrophs, contaminants and LAB. The third group of samples: increased variability	Not analyzed	133,331 reads passed filters, average value: 4597 reads/sample ESC > 99%	(Guidone *et al.* 2016)
Caciocavallo of Castelfranco cheese	raw cows' milk	Campania region	natural whey cultures	Biostic TM Bacteremia DNA isolation kit (MO BIO).	V1-V3 region of 16S rRNA	Hay (H): Cheese day 60: *Lactobacillus sp.* 6.29%, *L. casei*: 28.40%, *L. delbrueckii*: 0.68%, *S. thermophilus*: 62.13%<br>Silage (S): Cheese day 60: *Lactobacillus sp.* 3.55%, *L. casei* group: 9.38%, *L. delbrueckii*: 0.65%, *S. thermophilus*: 78.72%	Not analyzed	128,014 reads passed filters, average value: 7111.8 reads/samples ESC >90%.	(Giello, M *et al.* 2017)
Herve cheese	Raw / pasterized milk	Herve Belgium	Rennet, *Lactococcus lactis ssp. cremoris, Lactococcus lactis ssp. lactis,* Lactococcus lactis ssp. cremoris biovar diacetylactis, Leuconostoc spp.	DNeasy Blood & Tissue DNA extraction kit (Qiagen)	E9-29 and E514-530 (Brosius *et al.,* 1981)	Raw milk: Core: *L. lactis ssp. cremoris*: 79.5%), *Staphylococcus equorum*: 6.0%, *Psychrobacter celer:* 5.7%, *Streptococcus salivarius ssp. thermophilus:* 4.1%<br>Rind: *Corynebacterium casei*: 51.17%, *L. lactis ssp. cremoris:* 11.62%, *Staph. equorum*: 5.54%, *Marinilactibacillus psychrotolerans*: 4.55%, *Vagococcus salmoninarum*: 3.16%, *Psychrobacter spp.*: 2.94%, *Fusobacterium spp.*: 2.54%, *Clostridiisalibacter*: 2.48%, and *Strep. Salivarius*: 1.93%.<br>Pasterized milk: Core: *L. lactis ssp. cremoris:* 76.87%, *Psychrobacter sp.*: 11.35%, and *Staph. equorum*:6.57%<br>Rind: *Psychrobacter*: 37.76%, *L. lactis ssp. cremoris*: 10.76%, *C. casei:* 7.42%, *V. salmoninarum*: 5.69%, *Staph. equorum*: 5.28%, *Vibrio spp.*: 5.03%, *Fusobacterium spp.*: 4.39%, *M. psychrotolerans*: 3.69%, *Pseudoalteromonas* spp.: 2.71%, *Vagococcus fluvialis* 2.64%, and *Strep. salivarius:* 2.07%, *B. linens*: >2%	Not analyzed	62,218 reads, 2,828 mean number per sample	(Delcenserie V. *et al.* 2014)
**Sequencing platform: MiSeq, Illumina (San Diego, CA, USA)**									
Minas artisanal cheese	raw cow's milk	Serra do Salitre region,	endogenous starter culture	(Quigley *et al.* 2011).	V3/4/5 hypervariable region of bacterial 16S rRNA genes	Core: Dominant*: Streptococcus, Lactococcus, Lactobacillus*<br>Rind: *Streptococcus, Lactococcus, Staphylococcus, Weissella, Leuconostoc,* family Planococcaceae	Not analyzed	Mean 50.223 read counts per sample, 1.202 OTUs	(F. M. Sant'Anna et

		Minas Gerais state, Brazil	called “pingo”						al., 2019)
Reblochon-style cheeses’ rinds	pasteurized milk	France	*S.thermophilus, L. bulgaricus*, & ripening: *B. aurantiacum* & *D. hansenii G. candidum*	(Dugat-Bony *et al.* 2015)	V3V4 region of the 16S rRNA gene with the primers F343 and R784	*S. thermophilus*: 75.780%, *L. delbrueckii*: 11.029%, *L. casei*: 9.179%	Not analyzed	70 to 91 million sequencing reads generated from each of the 12 cheese rind samples	(Monnet *et al.* 2016)
Cantal cheeses	Cow milk	Marcenat France	*L.lactis, L. cremoris, L.mesenteroides*	mechanical lysis, phenol-based extraction.	V3-V4 of the 16S rRNA gene. Primers: PCR1F_460 and PCR1R_460	Core: *L. lactis*: 97.4%. Rind: *Brevibacterium linens*: 1.87%, *Brachybacterium paraconglomeratum/sacelli*: 1.22%, *L. lactis*: 69.7%	Not analyzed	A total of 1,636,464 reads from 48 samples, 365 OTUs	(Frétin *et al.* 2018)
Silter cheese	Raw cow milk	Brescia (Northern Italy)	*L. lactis, L. pseudomesenteroides L. mesenteroides, S. thermophilus*	(Cremonesi *et al.* 2006) with some modifications	V3-V4 of the 16S rRNA gene.	Dairy: R; with selected autochthonous starter: *Streptococcus*: 87.6%, *Leuconostoc*: 3.1 %, *Lactobacillus*: 6.47%, *Lactococcus*: 1%<br>Dairy: R; without the selected autochthonous starter: *Streptococcus*: 81.5%, *Lactococcus*: 7.7%, *Lactobacillus*:2.56%, *Leuconostoc*: 5.34%<br>Dairy: B; with the selected autochthonous starter: *Streptococcus*: 86.6%, *Lactococcus*: 1%, *Lactobacillus:* 13%, *Leuconostoc*: 2.76%<br>Dairy: B; without the selected autochthonous starter: *Streptococcus*: 80%, *Lactococcus*: 4.4%, *Lactobacillus:* 7.87%, *Leuconostoc*: 0.9%	Not analyzed	20.928.344 raw reads	(Silvetti *et al.* 2017)
semi-hard Swiss-type Maasdam cheese	pasteurized milk	Switzerland	*L.lactis and L.cremoris P. shermanii, L.rhamnosus L. helveticus as adjunct cultures.*	Wizard® genomic DNA Purification Kit (Promega)	Adaptors only	**Cold ripening**: *L. lactis*: 85.554%, *L. helveticus:* 11.375%, *P. shermanii*: 0.9%, *L. rhamnosus*: 1.05%, *S.thermophilus*: 1.2%, **Warm ripening**: *L. lactis*: 82.5%, *L. helveticus:* 10.5%	Not analyzed	17 million high quality reads were obtained	(Duru *et al.* 2018)
Serpa cheese	raw ewes' milk	Portugal	*dried flowers from Cynara cardunculus L. as rennet*	PowerFood Microbial DNA Isolation kit (MoBio USA)	F: ITS3NGS1, ITS3NGS2, ITS3NGS3, ITS3NGS4, ITS3NGS5, & ITS3NGS10. R: ITS3NGS001	Not analyzed	*Cryptococcus, Hanseniaspora, Saccharomyces, Kluyveromyces, Debaryomyces, Pichia, Metschnikowia, Yarrowia, Candida, Galactomyces, Penicillium, & Basidiomycota*	Not mentioned	(Gonçalves DSMTP *et al.* 2017)

Dutch-type cheese	Pasteurized milk	Netherland	*freeze-dried CH-N 19 starter*	UltraClean® GelSpin® DNA Extraction Kit (Mobio, USA)	V3 and V4 of the bacterial 16S rRNA gene. primers: Uni340F & Bac806R	99.9%: *Lactococcus (L. lactis/cremoris, L. raffinolactis* & *L. chungangensis*), *Leuconostoc* (*L. mesenteroides* & *pseudomesenteroides)* and *Lactobacillus (*L. *curvatus/sakei* & Lactobacillus spp.	Not analyzed	2,905,499 high quality sequences, 69 OTUs	(Porcellato *et al.* 2016)
Tomme d'Orchies	Cow raw milk	France	*PAL114D (L. lactis, L. cremoris, L. lactis biovar diacetylactis, S.thermophilus, Lb. helveticus, Lb.lactis), Propionibacterium PAL25-2 (P. freudenreichi subsp. shermanii) and D. hansenii (PAL DH10)*	DNeasy Blood & Tissue DNA extraction kit	V1–V3 hypervariable regions, forward (5'CGGCAGCGTCAGATGTGTATAAGAGACAG-3') and reverse (5'-GTVTVGTGGGCTCGGAGATGTGTATAAGAGACAG-3')	Core: Day 0*: Lactococcus lactis*: 30.97%, *Lactococcus cremoris*: 24.96%, *Lactococcus sp.*: 10.16%, *Streptococcus thermophilus:* 25.17%, *Streptococcus* sp. 4.30%.<br>Day 21: *Lactococcus lactis*: 6.28%, *Lactococcus cremoris*: 1.96%, *Streptococcus thermophilus:* 78.71%, *Streptococcus* sp. 9.58%.<br><br>Surface: Day 0*: Lactococcus lactis*: 12.29%, *Lactococcus cremoris*: 2.60%, *Lactococcus sp.*: 4.83%, *Corynebacterium variabile*: 8.74%, *Corynebacterium flavescens*: 4.36%, *Corynebacterium* sp.2.84%, *Lactobacillus helveticus*: 10.76%, *Lactobacillus capillatus*: 3.54%, *Lactobacillus rhamnosus*: 1.23%, *Lactobacillus* sp.: 8.51%, *Streptococcus thermophilus:* 8.24%, *Streptococcus* sp. 1.97%, *Brevibacterium linens*: 3.68%.<br>Day 21*: Corynebacterium variabile*: 19.76%, *Corynebacterium flavescens*: 1.32%, *Corynebacterium casei*: 1.41%, *Corynebacterium* sp.: 3.93%, *Lactobacillus* sp.: 1.15%, *Brevibacterium linens*: 1.56%, *Leucobacter chromiireducens*: 5.46%, *Peptostreptococcus russelli*: 4.02%, *Psychrobacter celer*: 12.98%, *Psychrobacter* sp.: 12.46%	Not analyzed	Not mentioned	(Ceugniez A.*et al.* 2017)
Plaisentif	Cow raw milk	Piedmont region, Italy	*no*	Dneasy Blood & Tissue kit (Qiagen)	V4 region of the bacterial 16S rRNA gene, universal primers 515F and 806R	*Lactococcus, Lactobacillus* and *Streptococcus* (40% and more than 90%), *Leuconostoc, Enterococcus, Acinetobacter, Chryseobacterium, Staphylococcus, Enhydrobacter, Sphingomonas, Bacillus, Corynebacterium, Pseudomonas.* (1%–0.01%), *Granulicatella, Brevibacterium, Salinicoccus, Vagococcus, Anaerobacillus, Sphingobacterium, Klebsiella, Carnobacterium, Pediococcus, Brachybacterium, Morganella Erwinia, Psychrobacter, Ralstonia, Veillonella, Cloacibacterium, Actinomyces, Flavobacterium, Capnocytophaga* (0.01%–0.0001%)	Not analyzed	2,819,261 high-quality 16S rDNA gene sequences, average sequence length of 252 bp	(Dalmasso et al., 2016)
**Sequencing platform: Illumina HiSeq 2000 (Harvard FAS Center for Systems Biology)**									
Cotija cheese	raw cow milk	*Mexico*	*no*	phenol-chloroform method	-	80%: *L. plantarum, L. mesenteroides* and *W. paramesenteroides*. More than 1% and less than 10%: *Aerococcus, Enterococcus, Lactococcus* and *Staphylococcus* (subdominant population).	Not analyzed	1,165,460 16S rRNA reads extracted	(Escobar-Zepeda *et al.* 2016)
**Sequencing platform: PacBio RS II (Pacific Biosciences, Menlo Park, CA)**									
Buryatia	raw cow milk	*Buryatia, Eastern Siberia*	*Handmade butter or cream*	(Yu *et al.*, 2009)	27F (5'-AGAGTTTGATCCTGGCTCAG-3'), 1495R (5'-CTACGGCTACCTTGTTA	Genera*: Lactococcus* (51.46%), *Streptococcus* (17.81%), *Pseudomonas* (5.48%), *Acetobacter* (4.83%), *Klebsiella* (3.36%), *Lactobacillus* (2.36%), *Acinetobacter* (1.84%), and *Raoultella* (1.63%). *Lc. lactis* (43.89%), *S. thermophilus* (8.52%), *Lactococcus raffinolactis* (6.12%), *Acetobacter cibinongensis* (4.80%),	Not analyzed	9,596.85 ± 2,512.28 high-quality sequences per sample	(Jin *et al.* 2018)

					CGA-3'), full length 16S rRNA region	*Lactobacillus helveticus* (1.95%), *Klebsiella pneumonia* (1.76%), *Acinetobacter johnsonii* (1.28%), and *Klebsiella oxytoca* (1.11%).			
